# Comparative analysis of sugarcane bagasse metagenome reveals unique and conserved biomass-degrading enzymes among lignocellulolytic microbial communities

**DOI:** 10.1186/s13068-015-0200-8

**Published:** 2015-02-08

**Authors:** Wuttichai Mhuantong, Varodom Charoensawan, Pattanop Kanokratana, Sithichoke Tangphatsornruang, Verawat Champreda

**Affiliations:** Enzyme Technology Laboratory, Bioresources Technology Unit, National Center for Genetic Engineering and Biotechnology (BIOTEC), Thailand Science Park, Pathumthani, 12120 Thailand; Department of Biochemistry, Faculty of Science, Mahidol University, Bangkok, 10400 Thailand; Integrative Computational BioScience (ICBS) Center, Mahidol University, Nakhon Pathom, 73170 Thailand; Genome Institute, National Center for Genetic Engineering and Biotechnology (BIOTEC), Thailand Science Park, Pathumthani, 12120 Thailand

**Keywords:** Lignocellulose degradation, Metagenomics, Comparative genomics, Biorefinery, Biofuels

## Abstract

**Background:**

As one of the most abundant agricultural wastes, sugarcane bagasse is largely under-exploited, but it possesses a great potential for the biofuel, fermentation, and cellulosic biorefinery industries. It also provides a unique ecological niche, as the microbes in this lignocellulose-rich environment thrive in relatively high temperatures (50°C) with varying microenvironments of aerobic surface to anoxic interior. The microbial community in bagasse thus presents a good resource for the discovery and characterization of new biomass-degrading enzymes; however, it remains largely unexplored.

**Results:**

We have constructed a fosmid library of sugarcane bagasse and obtained the largest bagasse metagenome to date. A taxonomic classification of the bagasse metagenome reviews the predominance of Proteobacteria, which are also found in high abundance in other aerobic environments. Based on the functional characterization of biomass-degrading enzymes, we have demonstrated that the bagasse microbial community benefits from a large repertoire of lignocellulolytic enzymes, which allows them to digest different components of lignocelluoses into single molecule sugars. Comparative genomic analyses with other lignocellulolytic and non-lignocellulolytic metagenomes show that microbial communities are taxonomically separable by their aerobic “open” or anoxic “closed” environments. Importantly, a functional analysis of lignocellulose-active genes (based on the CAZy classifications) reveals core enzymes highly conserved within the lignocellulolytic group, regardless of their taxonomic compositions. Cellulases, in particular, are markedly more pronounced compared to the non-lignocellulolytic group. In addition to the core enzymes, the bagasse fosmid library also contains some uniquely enriched glycoside hydrolases, as well as a large repertoire of the newly defined auxiliary activity proteins.

**Conclusions:**

Our study demonstrates a conservation and diversification of carbohydrate-active genes among diverse microbial species in different biomass-degrading niches, and signifies the importance of taking a global approach to functionally investigate a microbial community as a whole, as compared to focusing on individual organisms.

**Electronic supplementary material:**

The online version of this article (doi:10.1186/s13068-015-0200-8) contains supplementary material, which is available to authorized users.

## Background

Lignocellulose is a basic constituent of plant biomass and represents one of the most abundant sources of renewable carbon in the biosphere. Its complex structure consists mainly of carbohydrate polymers: cellulose, hemicellulose, and lignin. In nature, the degradation of lignocellulose requires multiple enzymes produced by diverse microorganisms, which act corporately and attack the complex structure of lignocellulosic biomass [1,2]. The growing number of studies on the complex pathways of lignocellulose degradation not only allows us to comprehensively understand the mechanisms and interplay between microbes in maintaining carbon balance in geobiochemical cycles, but may also lead to potential discovery of uncharacterized microbes and novel enzymes, which in turn, could improve the conversion of underused plant biomass to biofuels, chemicals, and other materials for biorefinery industries [3-7].

An industrial bagasse collection site at sugar mills represents a unique ecological niche for lignocellulose decomposition due to its high enrichment of lignocellulosic materials under high temperature and low nitrogen, with varying microenvironmental conditions, from the aerobic pile surface to the anoxic interior region. Recent studies have showed complexity in the bagasse microbial community and inherent metabolic potential in plant biomass decomposition, which provides novel genetic resources for biotechnological exploration [5,8,9]. However, it remains to be seen how the phylogenetic diversity and biomass-degrading enzyme repertoire of this microbial community compare to those of previously characterized lignocellulose-degrading environments.

Comparative metagenomic studies have been used to investigate the microbial communities in different environments in terms of taxonomy, gene contents, and also biochemical and metabolic potentials [10-14]. Culture-independent high-throughput sequencing has previously been used to explore the complexity of metagenomes obtained from several lignocellulose-degrading environments, including peat swamp forest [15], cow rumen [16,17] wallaby gut [18], and termite gut [19]. A comparison of soil metagenomes from distinct geographical locations, including cold and hot deserts, forests, grasslands, and tundra, has demonstrated the uniqueness of microbial communities in terms of taxonomic diversity and also the high relative abundance of functional genes that can be linked to the metabolic capability required to cope with specific environmental conditions [13]. Other comparative metagenomic analyses performed in different biomass-degrading environments also showed variation in metabolic potentials and enzymatic profiles related to decomposition of plant biomass in various ecological niches with different temperature, pH, and oxygen availability, for example, composts from a tropical zoo park [20], animal guts [21], and structurally stable symbiotic biomass-degrading consortia [22]. These findings thus shed light onto the highly complex mechanism of plant biomass decomposition through cooperative interactions between multiple microbial species and their enzymes in different environments. Metagenomic studies from biomass-degrading environments also serve as a useful starting point to discover new uncharacterized enzymes. As demonstrated in a study using ultra-deep sequencing of switchgrass degraded in cow rumens [17], only 12% of carbohydrate-active genes sequenced have 75% or more identity to known genes, suggesting a great potential of new enzyme discovery.

In this study, a fosmid library of a microbial community extracted from industrial sugarcane bagasse was constructed and analyzed by shotgun pyrosequencing to characterize and catalog the biodiversity of the microbe community, as well as its lignocellulolytic enzyme potential in biomass decomposition. The metagenome sequenced from this bagasse fosmid library, called the bagasse metagenome herein, was analyzed and compared with several reported metagenomic datasets from both lignocellulolytic and non-lignocellulolytic ecological niches. As well as elucidating the taxonomic compositions of microorganisms in different metagenomes, we have identified the biomass-degrading genes conserved among different microbial communities and the unique genes that could be related to specific metagenomes, thus providing a basis for understanding the roles and interplays of different microbes and their enzymes in biomass degradation in different environments.

## Results and discussion

### Constructing the fosmid library and pyrosequencing of sugarcane bagasse metagenome

We first constructed a fosmid library from the microbial DNA sequences obtained from the soil-contacting region of sugarcane bagasse collected from an industrial collection site (see Methods for more details). The fosmid clones were pooled and pyrosequenced on one full lane of a 454 Genome Sequencer FLX (Roche, Branford, CA, USA). Approximately one million raw reads were obtained, with an average read length of 570 bp (Table 1). Low quality sequences including short reads (<100 bp) and repetitive sequences were filtered out. The sequences contaminated by the vector and host genome used in the fosmid library construction were also removed at this step. After this data filtering, 726,980 reads remained with an average read length of 580 bp, and were subsequently assembled for longer overlapping sequences. This resulted in a total of 17,829 assembled contigs and 185,543 non-redundant singletons, which were then used for functional and comparative genomic analyses (see Additional file 1: Figure S1 for summary of data analyses). The entire bagasse metagenomic library has been deposited to the National Center for Biotechnology Information (NCBI) Sequence Read Archive (SRA) (SRX493840).

**Table 1:** **Summary of bagasse fosmid pyrosequencing data**

**Raw reads**						
**Dataset**	**Number of sequences**	**Number of nucleotides**	**Sequence length**			
			**Average**	**SD**	**Minimum**	**Maximum**
1. Raw reads	1,038,205	591,656,071	569.9	173.3	40	1,595
2. Read screen repeats	982,383	569,556,388	579.8	164.7	40	1,595
3. Read screen repeats and trim vector	726,980	421,491,438	579.8	166.0	40	1,595
**Assembled sequences**						
**Dataset**	**Number of sequences**	**Number of nucleotides**	**Sequence length**			
			**Average**	**SD**	**Minimum**	**Maximum**
1. Contigs	17,829	32,867,905	1,843.5	2,394.6	100	46,577
2. Singletons (non-redundant)	185,543	109,290,202	589.0	163.5	40	1,595

The bagasse fosmid library was sequenced on one full lane of the 454 GS-FLX Titanium, resulting in approximately one million raw reads. The reads with contaminating sequences of vector or host genome were removed before contig assembling and redundant sequence cleaning.

### Taxonomic classification and microbial diversity of the bagasse fosmid library

**Figure 1:**
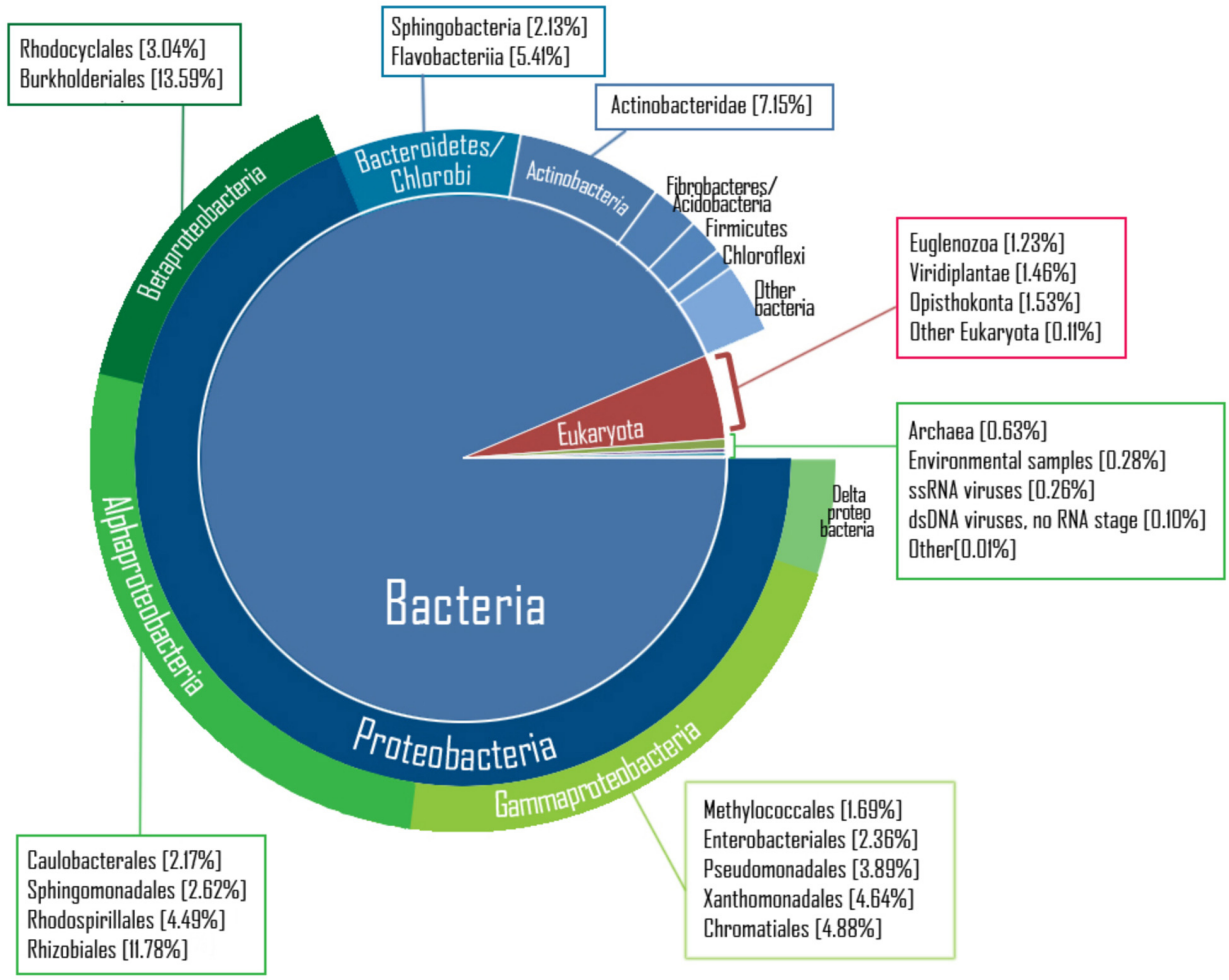
**Taxonomic distribution of a bagasse fosmid library.** A large majority of sequences in the library were classified as of bacterial origin (about 94%), followed by eukaryotes (about 4%) and archaea (about 0.5%). The taxonomic terms were obtained using BLASTN against non-redundant NT database using E-value cutoff at 1e-3. The pie chart represents percentages of reads that have the best hits (lowest E-value) to particular taxa.

To explore the phylogenetic diversity and complexity of the microbial community in the bagasse metagenome, we first identified taxonomic classification of singletons and contigs before removing duplicates, using BLASTN against the NCBI non-redundant nucleotide database (NT) [23] (Figure 1 and Additional file 2: Table S1). Based on the NCBI attributes, most of the mapped sequences from our bagasse fosmid library are of bacterial origin (94.4%, with a total of 1,164 assigned unique bacterial species), together with a small amount of eukaryotic DNA (4.3%), which are mainly from plants and fungi, and only trace of archaeal DNA (0.6%). We note; however, that functional genes of eukaryotes were predicted with a smaller degree of confidence than those of prokaryotes, as the genes are normally longer and frequently contain isoforms. The rest of the sequenced DNA comprises traces of DNA from viruses and other unidentified sequences.

Focusing on bacteria, the majority of reads were mapped to the Proteobacteria phylum (approximately two thirds of all mapped reads), which is metabolically versatile and spanned a wide range of bacterial taxa capable of aerobic as well as fermentative anaerobic metabolisms. The predominance of Proteobacteria in the sample collected from the exterior of a bagasse pile is in line with our previous observation in tagged 16S rRNA of the bagasse samples [5]. In our bagasse metagenome, most of the Proteobacteria have been assigned to one of three major classes: Alpha-, Beta-, and Gammaproteobacteria. Alphaproteobacteria is the largest class (22.5% of mapped reads) of microbes found in the bagasse metagenome, comprising both the aerobic and anaerobic bacterial orders Rhizobiales, Rhodospirillales, Sphingomonadales, and Caulobacterales. The majority of Gammaproteobacteria (19.1%) found belong to the Pseudomonadales, Chromatiales, Xanthomonadales, Methylococcales, and Enterobacteriales orders, whereas almost all Betaproteobacteria are from the genus *Burkholderia* (13.6%).

The next largest phyla are Bacteroidetes (10.2%) and Actinobacteria (7.9%), followed by relatively smaller amounts of DNA from Acidobacteria, Chloroflexi, and Firmicutes. Bacteroidetes are mostly anaerobic and are widely distributed in soil, sediment, aquatic habitats, and animal guts [6,24-27]. Actinobacteria are active biomass degraders under aerobic conditions and either mesophilic or thermophilic temperature ranges, and they have a significant role in lignocellulose decomposition in soil and aquatic environments [28,29].

### Biomass-degrading metabolic potential in bagasse fosmid library

**Figure 2:**
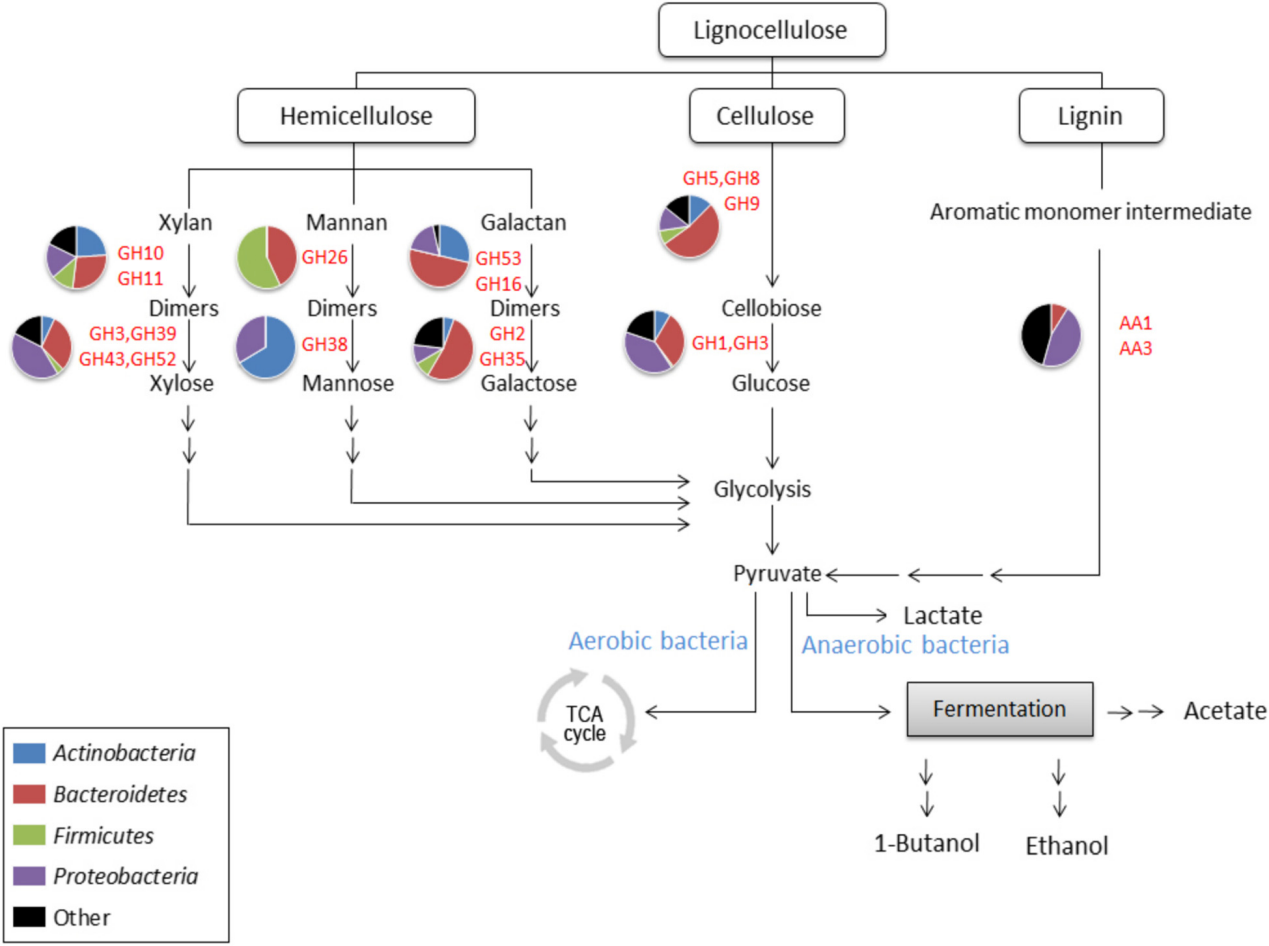
**Lignocellulosic degradation pathway and its related enzymes found in our bagasse metagenome.** Simplified biomass degradation process and enzymes involved. The enzyme families present in the bagasse metagenomic library are highlighted in red text. Colored pie charts show the amount of reads mapped to different GH families involving different steps of biomass degradation that belong to major bacterial phyla.

We then explored the repertoire of lignocellulose-degrading enzymes in the bagasse microbial community by assigning the predicted open reading frames (ORFs) with three carbohydrate-active enzyme families from the CAZy database [30]: glycoside hydrolases (GHs), carbohydrate-binding modules (CBMs), and the recently introduced auxiliary activities (AAs), to the non-redundant reads (see Methods). Of all the predicted ORFs, 1,774 (approximately 1%) have hits to 72 GH, 18 CBM, and 7 AA families (as summarized in Figure 2).

The microbial community found in bagasse is capable of producing various types of enzymes required to convert cellulose, hemicellulose, and lignin into different types of monosaccharides that are essential energy sources for aerobic (via the tricarboxylic acid, or TCA, cycle) as well as anaerobic bacteria (through fermentation processes). Of all the ORFs mapped to the GH families, 679 ORFs (about 42%) are related to 27 GH families that have lignocellulose-degrading enzymatic activities (Table 2). The majority of enzymes that degrade cellulose belong to two main families: GH5 and GH9, which contain cellulases including endoglucanases, exoglucanases, and beta-glucosidases. The exo-acting cellobiohydrolases are involved in initiating the attack on the highly ordered cellulose fraction comprising crystalline and amorphous regions. The cello-oligosaccharides and cellobiose are further processed by the enzymes involving the hydrolysis of beta-linked dimers of oligosaccharides such as beta-glucosidases from the GH1, 2, and 3 families.

**Table 2:** **Summary of the number of reads from the bagasse metagenome mapped to lignocellulose-degrading genes**

**Enzyme_group**	**Family**	**Actinobacteria**	**Bacteroidetes/ Chlorobi**	**Chlamydiae/ Verrucomicrobia**	**Chloroflexi**	**Fibrobacteres/ Acidobacteria**	**Firmicutes**	**Planctomycetes**	**Proteobacteria**	**Other** **bacteria**	**Other organisms**	**Activity**
Cellulases	GH5	6	18	1	0	3	4	0	4	0	0	Cellulase; endoglucanase; beta-glucosidase
Cellulases	GH6	3	0	0	0	0	0	0	0	0	0	Endoglucanase; cellobiohydrolase
Cellulases	GH9	1	9	0	0	0	0	0	2	3	1	Endoglucanase; beta-glucosidase
Cell wall elongation	GH16	8	12	0	1	0	0	0	5	0	0	Xyloglucan; licheninase
Cell wall elongation	GH17	0	0	0	0	0	0	0	28	0	0	Exo-beta-1,3-glucanase; licheninase
Cell wall elongation	GH74	0	0	0	0	0	2	0	0	0	0	Endoglucanase; xyloglucanase
Oligosaccharide-degrading enzymes	GH1	9	3	0	2	0	0	0	9	2	1	Beta-glucosidase; beta-galactosidase
Oligosaccharide-degrading enzymes	GH2	6	64	5	9	5	10	0	9	4	0	Beta-mannosidase; beta-galactosidase
Oligosaccharide-degrading enzymes	GH3	8	57	1	4	8	2	0	68	19	2	Beta-glucosidase; beta-glucosylceramidase
Oligosaccharide-degrading enzymes	GH29	0	14	1	0	7	4	1	0	0	0	Alpha-L-fucosidase
Oligosaccharide-degrading enzymes	GH35	1	0	3	1	0	0	0	3	0	1	Beta-galactosidase
Oligosaccharide-degrading enzymes	GH38	4	0	0	0	0	0	0	2	0	0	Alpha-mannosidase
Oligosaccharide-degrading enzymes	GH39	7	0	0	2	0	1	0	4	0	0	Beta-xylosidase
Oligosaccharide-degrading enzymes	GH42	0	0	0	0	0	0	0	0	2	0	Beta-galactosidase
Oligosaccharide-degrading enzymes	GH43	1	12	1	2	1	0	0	20	0	0	Beta-xylosidase
Oligosaccharide-degrading enzymes	GH52	0	0	0	0	0	5	0	1	0	0	Beta-xylosidase
Endohemicellulases	GH8	0	2	0	0	0	0	0	1	0	0	Endo-1,4-D-glucanase; chitosanase
Endohemicellulases	GH10	11	14	2	0	5	6	0	8	0	0	Xylanase; beta-1,4-xylanase; endo-1,4-beta-xylanase
Endohemicellulases	GH11	1	0	0	0	0	0	0	1	1	1	Endo-1,4-beta-xylanase; xylanase
Endohemicellulases	GH12	0	0	0	0	0	0	0	2	0	1	Endoglucanase; xyloglucan hydrolase
Endohemicellulases	GH26	0	3	0	0	0	4	0	0	0	0	Beta-mannanase; endo-1,4-beta-mannosidase
Endohemicellulases	GH28	0	15	0	0	4	9	0	2	4	0	Polygalacturonase; pectate lyase; endopolygalacturonase
Endohemicellulases	GH53	0	2	0	0	0	0	0	0	0	0	Endo-1,4-beta-galactosidase
Debranching enzymes	GH51	4	7	2	2	13	1	0	3	0	0	Alpha-L-arabinofuranosidase; endoglucanase
Debranching enzymes	GH62	2	0	2	0	0	0	0	1	0	0	Alpha-L-arabinofuranosidase
Debranching enzymes	GH67	0	6	0	0	0	0	0	3	0	0	Alpha-glucuronidase
Debranching enzymes	GH78	0	11	0	3	1	0	0	0	0	0	Alpha-L-rhamnosidase

Summary of the number of reads in the bagasse metagenome mapped to genes encoding lignocellulose-degrading enzyme homologs annotated by the CAZy database.

Hemicellulose contains a greater variety of carbohydrate compositions and thus requires a broader range of endo-acting enzymes to degrade, including endo-1,4-beta-xylanase (GH10) for hydrolysis of xylan, the most abundant hemicellulose in bagasse; endo-1,4-beta-mannosidase (GH26) for mannan; and endo-1,4-beta-galactosidase (GH16) for galactan. The genes encoding these enzymes are all present in the bagasse metagenome. The higher percentage of reads mapped to GH10 (2.6%) might reflect the requirement to digest the greater abundance of xylan, as compared to mannan (GH26, 0.4%) and galactan (1.5%). Further downstream, xylan is degraded by xylo-oligosaccharide hydrolyzing enzymes and side chain cleaving hydrolases such as beta-glucosidases and beta-xylosidases from multiple families, including GH3, 39, 43, and 52. The downstream decomposition of manno-oligosaccharides and their dimeric sugars is catalyzed by alpha-mannosidase (GH38), whereas the dimeric sugars from galactan are degraded by beta-galactosidases from GH2 and 35. We also observed a wide range of debranching enzymes such as alpha-L-arabinofuranosidase (GH62), alpha-glucuronidase (GH67), and alpha-L-rhamnosidase (GH78, relating to pectin degradation), reflecting corporate enzymatic functions in the degradation of hemicellulose.

In addition to a variety of glycoside hydrolases that degrade cellulose and hemicellulose, we also observed a moderate fraction of ORFs mapped to carbohydrate-binding modules (CBMs), a group of non-catalytic proteins that promote the association of the enzymes and substrates. The majority of the CBM enzymes found in the metagenome were identified as CBM50 (24.2%) and CBM48 (17.4%), peptidoglycan-binding and glycogen-binding proteins, respectively, although they are not known to be directly related to lignocellulosic hydrolysis. We also found a number of a recently defined CAZy class of lignin-degrading enzymes known as auxiliary activities (AAs), which contains eight families of ligninolytic enzymes and three families of lytic polysaccharide mono-oxygenases. The majority of lignin-breakdown enzymes found are multicopper oxidase (AA1, 25.0% of all AAs) and choline dehydrogenase (AA3, 30.0%), followed by smaller amounts of AA4, 5, 6, 7, and 9.

In terms of the microorganisms producing carbohydrate-degrading enzymes, our results show that heterogeneous hemicellulose and cellulose are degraded by specific endo-acting enzymes produced by all major bacterial phyla: Actinobacteria, Bacteroidetes, Firmicutes, and Proteobacteria (Figure 2), except for mannan, which specifically requires the GH26 (beta-mannanase) family from Bacteroidetes and Firmicutes. Mannobiose is then broken down into a single-molecule sugar by GH38 (alpha-mannosidase) from Actinobacteria and Proteobacteria. Other oligodimers from hydrolysis of lignocelluloses are subsequently degraded by specific exo-acting oligosaccharide-degrading and debranching enzymes produced from Actinobacteria, Bacteroidetes, and Proteobacteria, mainly from the bacterial orders of Sphingobacteriales, Actinomycetales, Caulobacterales, Cytophagales, and Ignavibacteriales. Lignin-degrading enzymes, on the other hand, are mostly generated by Bacteroidetes and Proteobacteria.

Proteobacteria is the most abundant phylum in the sugarcane bagasse community; however, smaller phyla such as Actinobacteria, Bacteroidetes, and Firmicutes contribute as much to the production of lignocellulolytic enzymes for the microbial community. Bacteroidetes, for instance, is the second largest bacterial phylum in our bagasse metagenome based on the number of mapped reads (10.2%), but is still far behind the most abundant phylum, Proteobacteria (66.1%). Intriguingly, the phylum Bacteroidetes produces the largest repertoire of many carbohydrate-degrading enzymes, especially cellulases (GH5, 9), oligosaccharide-degrading enzymes (GH2), and endo-hemicellulases (GH10, 28) (Table 2). This illustrates a complex interactivity and synergism of the microbial community in the decomposition of lignocellulosic biomass in the environment.

### Comparative genomic analysis of lignocellulolytic and non-lignocellulolytic metagenomes

**Figure 3:**
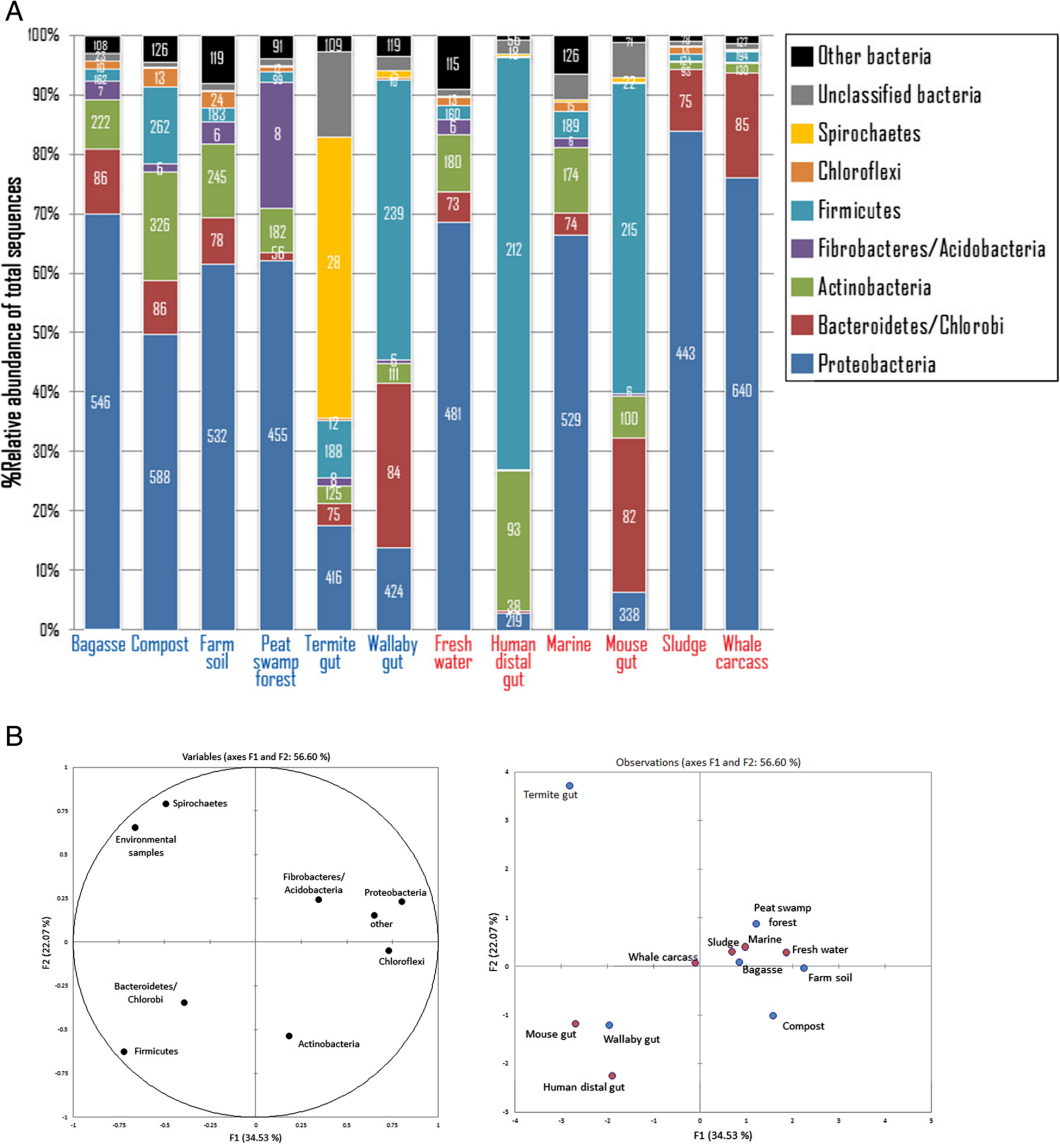
**Taxonomic profiles of metagenomes of lignocellulosic (blue)- and non-lignocellulosic (red)-degrading sources. A)** The relative taxonomic distributions of bacterial phyla in different metagenomic datasets. Each bar represents the percentage of total reads. The numbers within the bars indicate numbers of unique genes that reads from metagenomic libraries mapped to. Proteobacteria dominate almost all metagenomic communities, except for human gut, mouse gut, and wallaby gut, which are dominated by Firmicutes, and termite gut by Spirochaetes. **B)** Principal component analysis (PCA) of bacterial diversity profiles (left) and metagenome profiles (right). The bacteria of many phyla are found in highly overlapping environments, except for Firmicutes and Spirochaetes, which are predominantly present in mammal and termite guts; this explains why their profiles are not clustered with other metagenomes in the PCA plot.

Having explored the newly assembled metagenome from the sugarcane bagasse fosmid library, we now seek to investigate the shared and unique characteristics of the bagasse microbial community with other publicly available metagenomes. We have selected representative metagenomes from five other lignocellulolytic and six non-lignocellulolytic environments available from the NCBI Whole Genome Shotgun (WGS) and Sequence Read Archive (SRA) projects [31], based on comparable numbers of sequences and average lengths (Additional file 3: Table S2). The average number of reads is 98,000; the largest is approximately 200,000 reads (sugarcane bagasse from this study, and compost [32]), and the smallest is about 25,000 reads (human distal gut [33] and sludge [34]). The average read length of the combined dataset is approximately 1,000 bp. To minimize a potential bias from different analytic strategies previously used by different groups, we obtained assembled reads for each dataset and reanalyzed them using the same pipeline, as used in our sugarcane bagasse dataset (Additional file 1: Figure S1). We summarize the bacterial taxonomic distributions, which represent the largest superkingdom by far in these 12 metagenomes, in Figure 3 and Additional file 4: Table S3.

In brief, bagasse pile, farm soil, and compost are classified as active lignocellulose-degrading environments, comprising both aerobic and anoxic regions [5,32]. Decomposition of lignocellulose in bagasse in an open field is a slow process characterized by its low nitrogen, low moisture, and relatively high temperature conditions [5,8]. Farm soil is a complex and nutrient-rich environment active in decomposition of plant biomass and a wide range of organic materials [14]. The compost system is considered a relatively aerobic environment with dominant microorganisms in multi-phase composting processes, in which the highest biomass decomposition activity is found at the high-temperature thermophilic phase [32,35,36]. The gut ecosystems are closed anaerobic systems. Termite and wallaby guts represent examples of animal guts that are highly capable of digesting plant cell walls, and are thus considered as anaerobic lignocellulolytic environments [18,19]. However, we classified human and mouse guts as non-lignocellulolytic metagenomes in this study because the environments are relatively less effective in digesting a large amount of lignocellulosic biomass, although some symbiotic bacteria that produce polysaccharide-degrading enzymes can be found [25,37,38]. The rest of the metagenomes are considered inactive lignocellulose-degrading ecological niches under various physical and environmental conditions.

We first assessed the diversity of the above-mentioned microbial communities using the Shannon diversity index, based on 16S rRNA extracted from the metagenomes. The bagasse metagenome has a Shannon index of 2.08, comparable to the average of 2.76 ± 0.73 (SD) (Additional file 3: Table S2). There are 1,164 different bacterial species detected in the bagasse metagenome, whereas the average is 1,035.25 ± 201.45 (SD). Using the combined dataset from all 12 metagenomes as a reference, “all-combined” dataset herein, we observed that the microbial community in bagasse is more enriched in Proteobacteria than the average (all *P*-values < 2.2×10^-16^, Fisher’s exact test, unless indicated otherwise). This is still true even when compared with other lignocellulolytic datasets combined (Additional file 5: Table S4). By contrast, the bagasse metagenome has smaller proportions of reads identified as Actinobacteria, Cyanobacteria, and Firmicutes than the all-combined and lignocellulolytic datasets.

### Proteobacteria dominates “open environment” lignocellulolytic and non-lignocellulolytic metagenomes

Overall, the 12 microbial communities analyzed contain different combinations and abundances of the bacterial phyla, with five or more phyla in all the datasets analyzed (Figure 3A). Note that we present and discuss the abundances of reads belonging to taxonomic groups or functional categories as percentages of mapped reads in that dataset, as well as numbers of unique species or genes that any read in the dataset mapped to. The most distinguishable characteristic in the taxonomic profile is the prevalence of reads assigned to Proteobacteria between oxygenated “open” environments (for example, sugarcane bagasse, compost, and sludge) and anoxic “close” environments (such as animal guts). We observed the domination of reads from aerobic Proteobacteria in all the open-environment metagenomes, which account for more than half of all the mapped reads, whereas they are almost entirely absent from the metagenomes of animal guts, in agreement with previous studies [5,22]. Of all the four gut metagenomes included in this study, the animal guts that have relatively less effective cellulose-degrading function (human and mouse) predominantly contain anaerobic Firmicutes such as Clostridia, which have been shown to possess a number of lignocellulose-degrading genes [25,37-39]. Interestingly, the two herbivorous guts (termite and wallaby) contain different compositions of bacterial phyla. In the termite gut metagenome, approximately half of the sequenced reads were mapped to only 28 unique Spirochaetes species, whereas less than 20% of reads were mapped to 416 unique Proteobacteria, possibly because Proteobacterial genes are better characterized. By contrast, the majority of bacterial sequences in the wallaby gut belong to the Firmicutes phylum, similarly to what is observed in human and mouse guts. This is in line with earlier studies showing that gastrointestinal microbes of warm-blooded animals mainly comprise Firmicutes and Bacteroidetes [24,40,41].

We then performed a principal component analysis (PCA) to quantitatively assess the similarity between the presence/absence patterns of bacterial phyla in different environments (Figure 3B left), as well as between metagenomic datasets that contain different bacterial constitutions using a loading plot (Figure 3B right). The PCA result supports our previous observation that the three mammal gastrointestinal environments share similar sets of bacterial phyla compositions, which are mostly anaerobic. The termite gut, by contrast, has a unique composition of microbes, most likely due to a much higher pH environment [42]. This is also reflected by the distinct prevalence of anaerobic Spirochaetes, which are mostly found in the termite gut, but almost entirely disappear from other guts and in the open environments. Our comparative genomic analysis thus demonstrates that lignocellulosic and non-lignocellulosic biomass-degrading lifestyles are not necessarily linked to the taxonomic diversity of the microbial communities. For comprehensive analyses of genomes and their functions across multiple gastrointestinal metagenomes, we refer the reader to an earlier comparative genomic study [21].

### Meta-analysis of functional gene contents reviews high abundances of metabolic genes in biomass-degrading metagenomes

**Figure 4:**
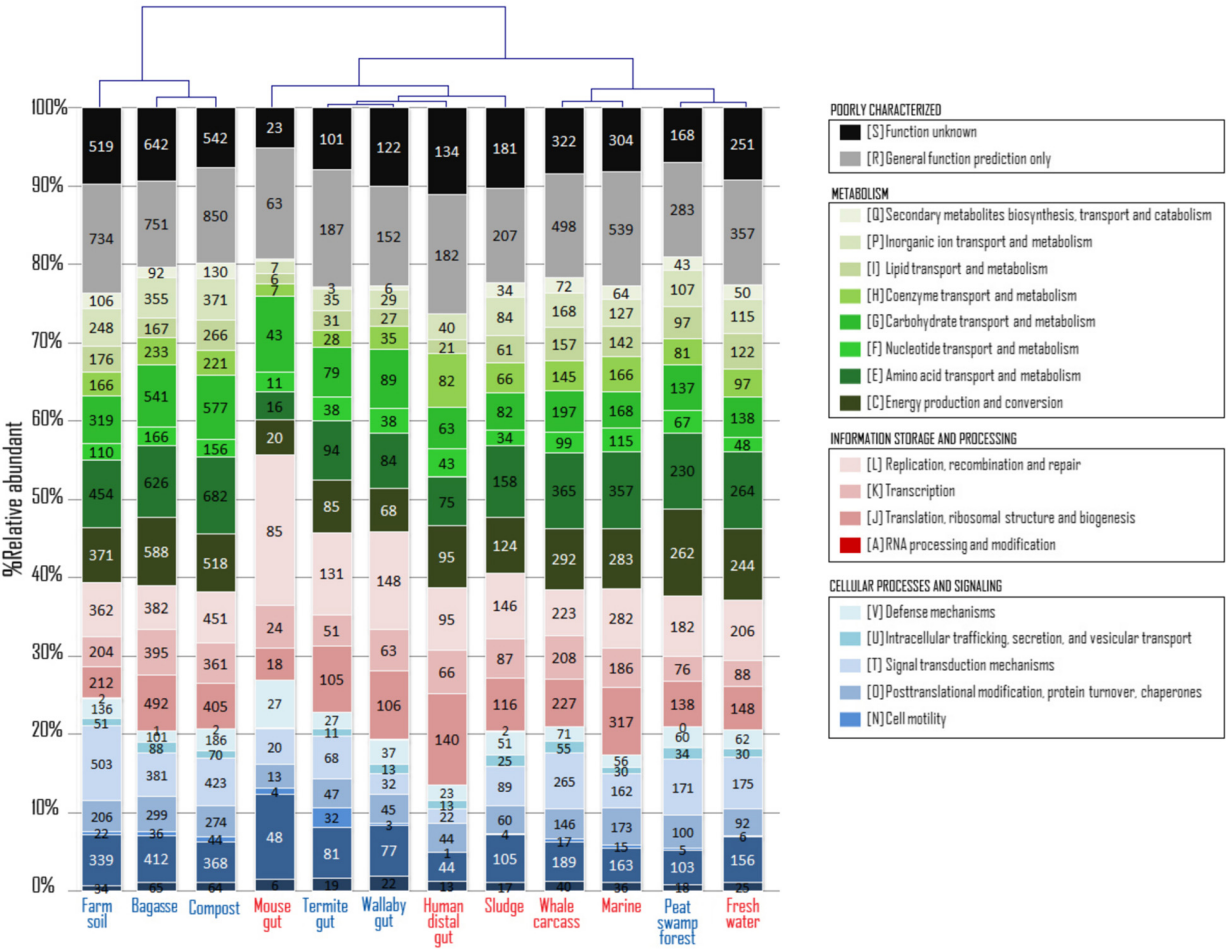
**Comparison of Clusters of Orthologous Groups (COGs) in lignocellulolytic and non-lignocellulolytic metagenomes.** The bar plots represent the percentage of reads mapped to different COGs using BLAST, while the numbers within the bars indicate the number of unique genes. Metagenomic profiles are clustered using hierarchical clustering (complete linkage method), based on the divergence of COG profiles.

To gain an overall picture of the functions of genes possessed by the 12 metagenomes, we first classified the assembled reads to different Clusters of Orthologous Groups (COGs) [43] (Figure 4 and Additional file 4: Table S3). Globally, we observed a large proportion (approximately 40% or more) of reads mapped to genes involving metabolic processes (green bars) in all the open aerobic environments, especially energy production and conservation [COG class C] and amino acid transport and metabolism [E]. However, metabolic COGs are present in only 20 to 30% of the reads from the animal gut metagenomes, which are hierarchically clustered together. As expected, bagasse and other lignocellulolytic metagenomes are more enriched in carbohydrate transport and metabolism [G] genes than the all-combined dataset, with the exception of wallaby guts (Additional file 5: Table S4). Interestingly, the majority of DNA sequences from the gastrointestinal metagenomes were mapped to the information storage and processing (red) genes, particularly replication recombination and repair [L] and translation, ribosomal structure, and biogenesis [J], and cellular processes and signaling (blue) genes, especially cell wall/membrane/envelope biogenesis [M]. The dominance of information and signaling genes is most pronounced in the mouse gut, where more than half of the mapped ORFs are involved in these two classes combined. The mouse gut also possesses a twofold higher amount of replication recombination and repair [L] genes than average (*P* < 10^-40^, Additional file 5: Table S4). The gut microenvironments are anoxic and nutrient-rich, and can have extremely low or high pH and temporal fluctuation of feces [40,42,44]. This might impose additional metabolic activities that require a large number of signaling and regulatory genes to help maintain homeostasis of cells in these unique environments [45-47].

Focusing on the number of unique genes that assembled reads were mapped to (indicated by the numbers within the bars), many metabolic COGs including the carbohydrate transport and metabolism [G] genes are most enriched in three lignocellulolytic environments: compost (577 unique genes), bagasse (541), and farm soil (319), suggesting a greater diversity of carbohydrate-active genes in these three metagenomes. However, one might consider that the numbers of total reads in these datasets are slightly larger than in other datasets (Additional file 3: Table S2), and thus contribute to the larger numbers of unique genes observed. We believe this is only partly true, as the number of reads from the peat swamp forest dataset is as large, but the numbers of unique genes are similar to those from the datasets with lower numbers of total reads.

We then focused on metabolic potential of the metagenomes using the Kyoto Encyclopedia of Genes and Genomes (KEGG) pathways [48] (Additional file 4: Table S3 and Additional file 6: Figure S2). All the KEGG classes involved in carbohydrate metabolism can be found in all the 12 metagenomes, and this is also true for most of the enzymes related to the metabolism of amino acids, and cofactors and vitamins. The gastrointestinal tract environments are, again, more similar to one another. We observed a number of KEGG classes specifically absent or present at much lower percentages in animal guts, including lipid metabolic classes such as alpha-linolenic acid metabolism and fatty acid elongation, whereas sphingolipid metabolic genes are more pronounced (Additional file 4: Table S3). Looking at the number of unique genes mapped to the KEGG carbohydrate metabolism pathways, a wide range of carbohydrate metabolism enzymes are detected in all the datasets impartially, except for the termite gut, which seems to have a lower number of enzymes than other datasets.

### Lignocellulolytic environments are enriched in enzymes required for degrading large carbohydrate molecules

So far we have addressed the taxonomic and functional genomic similarities between the sugarcane bagasse and other selected lignocellulolytic and non-lignocellulolytic metagenomes. In this section, we focus on the carbohydrate enzyme classifications from the CAZy database [30], which provides manually curated information for all characterized carbohydrate-active enzymes, covering cellulases, hemicellulases, and pectinases.

**Figure 5:**
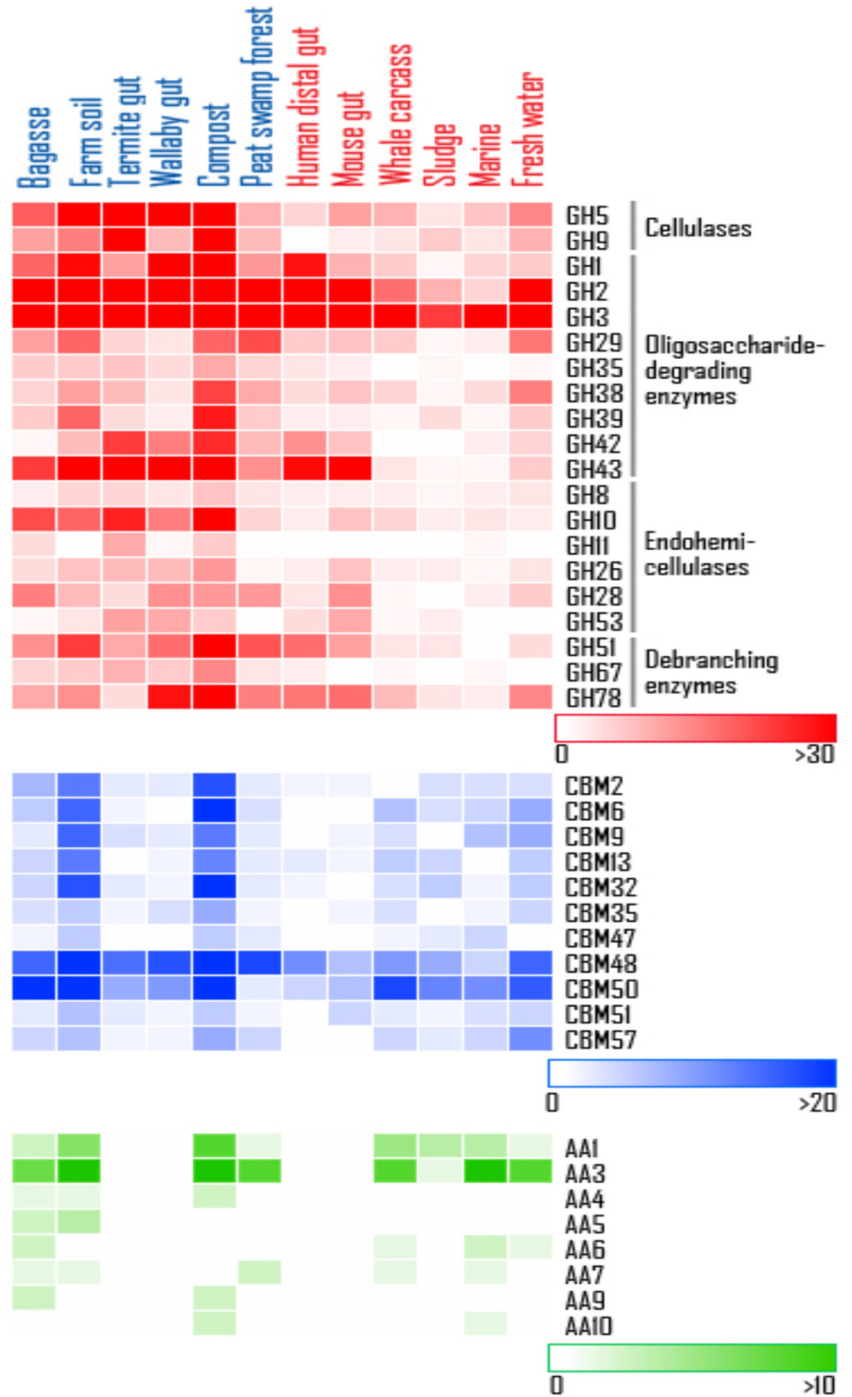
**Comparison of reads mapped to different KEGG metabolic pathways and CAZy enzyme families.** Comparative genomic analysis of selected glycoside hydrolase (GH), carbohydrate-binding module (CBM), and auxiliary activities (AA) families. Color shades indicate the numbers of unique genes in the families to which metagenomic reads were mapped.

As expected, lignocellulolytic metagenomes contain a greater number of unique GH genes with lignocellulose-degrading enzymatic activities (Figure 5, red), as well as the numbers of reads mapped to these GH families (Additional file 4: Table S3), especially those encoding enzymes acting on large carbohydrate molecules, further up the lignocellulose degradation pathway (see Figure 2 for a simplified pathway). To illustrate the point, the lignocellulolytic metagenomes have a larger repertoire of the so-called “true cellulases” (GH5, 9), as the numbers of unique genes are markedly higher than those of the non-lignocellulolytic metagenomes. Interestingly, although the lignocellulolytic metagenomes contain similar numbers of unique GH5 and 9 genes, they are most enriched in the termite gut in terms of read abundance (ninefold and fivefold of the all-combined dataset, respectively, *P* < 10^-19^ Additional file 5: Table S4). Similarly, several endo-acting hemicelluloses including GH10, 16, 26, 51, and 53 are all more abundant in the lignocellulolytic metagenomes based on unique genes as well as mapped reads, whereas GH11, a xylanase family, is almost entirely absent from the non-lignocellulolytic environments. However, major oligosaccharide-degrading families such as GH2 and 3, which are required at the later stage to break down disaccharides into monosaccharides, are present in nearly all the metagenomes analyzed at comparable gene numbers and percentages, with the exception of sludge, marine, and carcass, where GH2 is present at relatively lower abundances (*P* < 10^-19^ Additional file 5: Table S4). This suggests a remarkable ability of the microorganism communities in lignocellulolytic metagenomes to break down large carbohydrate molecules. Accessory enzymes involved in cleavages of hemicellulose side chains, for example, beta-galactosidases and alpha-arabinofuranosidases (GH43), are found in all open lignocellulolytic environments and in the guts of herbivores and omnivores. In addition to these lignocellulolytic “core” enzymes, we also observed a number of GH families most pronounced in bagasse, namely GH15 (glucoamylase), GH17 (glucan endo-1,3-beta-glucosidase) GH65 (maltose phosphorylase, rehalose phosphorylase), and GH115 (xylan alpha-1,2-glucuronidase) (*P* < 10^-3^ when compared with the all-combined dataset).

In a similar manner to the majority of the GH families, CBMs (for example, CBM2, 6, 9, and 32) are evidently most enriched in the farm soil and compost metagenomes, with the exception of CBM48 and 50, which are highly present in all the environments analyzed (Figure 5, blue). Note that although the numbers of raw reads and mapped ORFs obtained from the farm soil and compost environments are higher than the average of the 12 metagenomes, these numbers are still comparable to those of the bagasse and peat swamp metagenomes. The CBM2 family has been shown to bind to cellulose and xylan, while CBM9 is found only in xylanase. CBM6 has been demonstrated to function in binding of amorphous cellulose and xylan, as well as beta-1,3 and beta-1,4 glucan. A high abundance of CBM32, involved in binding to galactose and lactose, is also found in these two environments. The CBM48 family, on the other hand, binds to GH13, a large GH family that includes alpha-amylase, which is also highly enriched in both groups of environments. Similarly, CBM50 binds to a number of GH families including GH23, 24, 25, and 73, which are all found in both lignocellulolytic and non-lignocellulolytic metagenomes.

The AA class represents families of ligninolytic enzymes and lytic polysaccharide mono-oxygenases [30]. As lignin is intimately associated with the carbohydrates in the plant cell wall, these ligninolytic enzymes cooperate with the classical GHs in decomposition of lignocelluloses. Intriguingly, the AA families are absent from animal guts altogether (Figure 5, green). The two major families AA1 and 3, for instance, are present in all the microbial communities, except for the closed anaerobic environments, possibly because most AAs identified to date are related to aerobic fungi and bacteria. Importantly, the bagasse microbial community had the most complete set of AA families (seven out of eight families analyzed: AA1, 3, 4, 5, 6, 7, and 9). AA9 (formerly GH61) in particular, has received growing attention recently, as the remarkable synergism between AA9 and GHs in boosting enzymatic cleavages of lignocellulosic biomass has been reported and patented [49-52]. The AA9 proteins are copper-dependent lytic polysaccharide monooxygenases (LPMOs), which function in cleaving cellulose chains with oxidation of various carbons (C-1, C-4, and C-6) [53]. The AA9 family found in the bagasse metagenome originates from fungi, as in compost, the only other metagenome in this study where AA9 is found.

**Figure 6:**
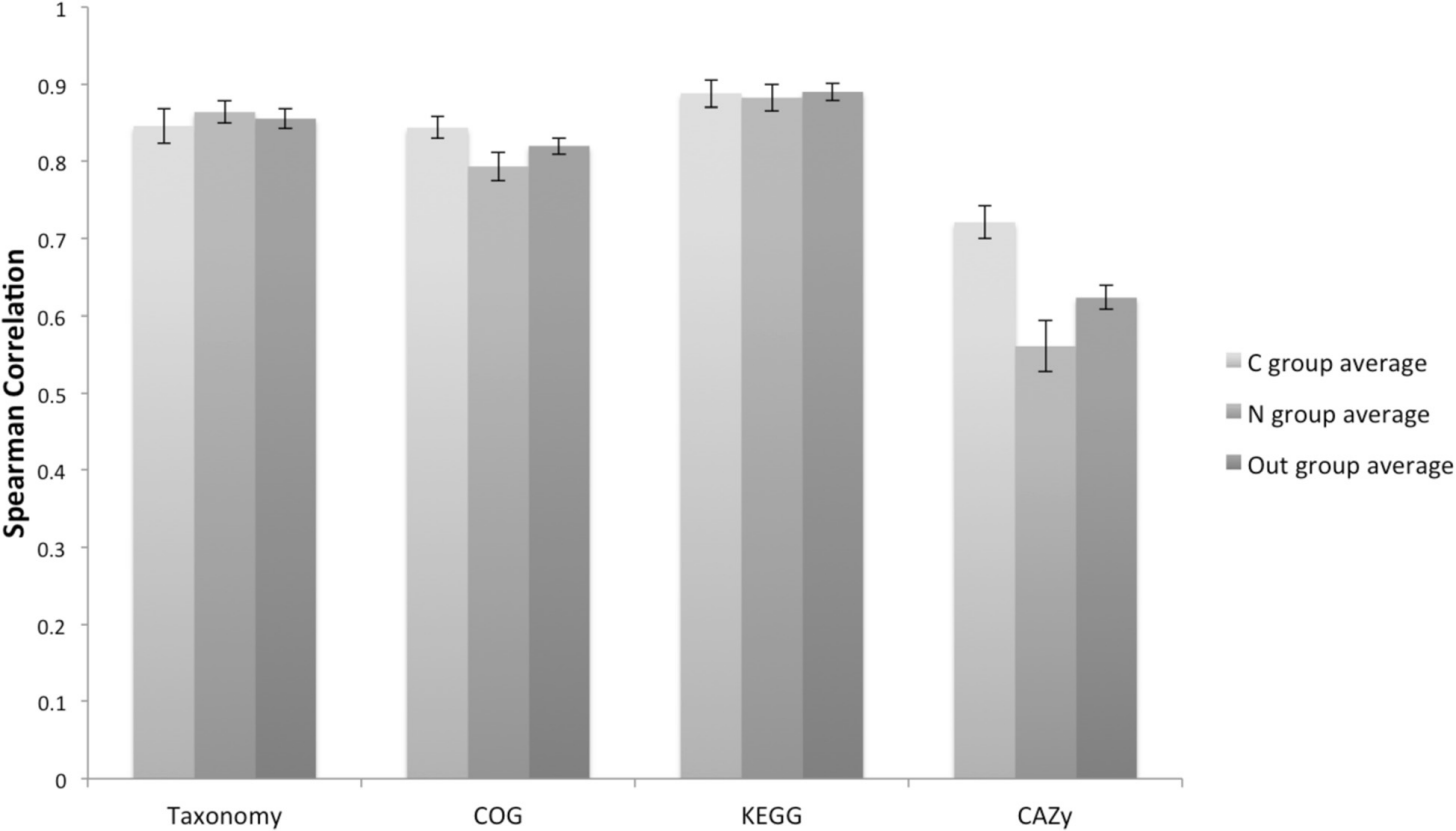
**Lignocellulolytic metagenomes are taxonomically diverse, but their carbohydrate-active enzymes are conserved.** Spearman correlations were computed for metagenomic libraries within the lignocellulose-degrading environment group (C), non-lignocellulose-degrading group (N), or metagenomes from different groups (Out group), based on the taxonomy, COG, KEGG, and CAZy profiles. Error bars represent standard errors of means.

To quantify the similarity between different metagenomic profiles, we have computed Pearson and Spearman correlations among all the metagenomes based on the three patterns of the four characteristics: taxonomic, COG, KEGG, and CAZy profiles (Figure 6 and Additional file 7: Table S5). As described in the previous sections, Figure 6 recapitulates our observation that the two groups of selected metagenomes: lignocellulolytic and non-lignocellulolytic, are hardly distinguishable based on the taxonomic, COG, or KEGG profiles. However, here we show that the lignocellulolytic metagenomes possess more similar sets of CAZy families, and also a significantly greater similarity of proportions of reads mapped to different families, than those within the non-lignocellulolytic group and also those between the metagenomes from different groups. The lignocellulolytic group also possesses a larger number of unique CAZy genes (1,118.5 ± 538.0, SD, 931 in bagasse) compared to the non-lignocellulolytic group (501.8 ± 140.8, SD). This signifies the common carbohydrate-degrading gene repertoire and composition in the lignocellulolytic metagenomes, which enable the microbial communities as a whole to harvest energy and nutrients from lignocellulosic biomass, regardless of the taxonomy and enrichment of individual organisms in each microbial community.

## Conclusions

Sugarcane bagasse is one of the most abundant agricultural biomasses, with a global production of over 250 million tons per year [5,54]. Microbial communities in industrial bagasse piles provide a useful starting point for the exploration and characterization of new biomass-degrading enzymes, which are stable and active at relatively high temperatures, in low amounts of nitrogen, and under the varying microenvironmental conditions commonly found in different regions of the piles. To the best of our knowledge, the phylogenetic distribution of microorganisms in the bagasse metagenome has previously been characterized using 16S rRNA and a restricted number of shotgun sequencing reads (70,000 reads) [5,8], and thus the metagenome constructed from the fosmid library in this study provides the largest collection of metabolic genes found in this ecological niche to date (approximately one million raw reads and over 200,000 assembled contigs plus singletons). This, for the first time, allows us to investigate the functions, prevalence, diversity, and abundance of biomass-degrading enzymes, all of which was not possible with the previous 16S rRNA and smaller shotgun sequencing libraries. The bagasse formid library also serves as a useful resource for subsequent enzymatic assays of prospective biomass-degrading enzymes, which could be developed further for industrial use. The fosmid library also allows easy recovery of genes of interest for further cloning and detailed expression study [5,55].

We have characterized our newly constructed bagasse metagenome and demonstrated that the microbe community is taxonomically diverse, and at the same time, functionally capable of converting large polysaccharides to monomeric sugars, as all the major cellulolytic and hemicellulolytic enzymes were found. Based on our comparative genomic analyses of the bagasse metaganome with other publicly available lignocellulolytic and non-lignocellulolytic metagenomes, we have shown that the phylogenetic distributions of the microbes are separated mainly by their aerobic/anoxic lifestyles. Intriguingly, although the lignocellulolytic and non-lignocellulolytic groups are not distinguishable by their taxonomic contents or by their high-level functional classifications (COGs) and metabolic genes (KEGG), the lignocellulolytic group possesses highly similar lignocellulose-degrading core genes, which are produced by different types and abundances of microbes within different lignocellulose-degrading communities. That is, even though the species compositions of lignocellulolytic metagenomes are no more similar than when they are compared across the two groups, their carbohydrate-active enzyme compositions are significantly more conserved than those in non-lignocellulolytic groups. This exemplifies an important interplay between diverse microorganisms in the communities that contribute to the enzyme repertoires required to degrade lignocelluloses under mixed microenvironmental conditions, in different ecological systems.

## Methods

### Sample collection and DNA extraction

The sugarcane bagasse sample was collected from soil-contacting regions of a bagasse pile (one meter from the edge of the pile) at Phu Khieo Bio-Energy Chaiyaphum province, Thailand (N 16°28’54”, W 102°07’05”). The bagasse piles are normally approximately 10 m in height covering an area of several acres. The sample was rapidly frozen in liquid nitrogen and kept at -80°C for subsequent experiments. Metagenomic DNA was directly extracted from a sample by the SDS-based DNA extraction procedure [56], with slight modifications [57]. Briefly, five grams of sample was subjected to direct cell lysis with DNA extraction buffer, proteinase K, and sodium dodecyl sulfate (SDS). Protein contamination was removed by chloroform extraction, and then the DNA was precipitated with isopropanol. High molecular weight DNA of size ranging from 30 to 50 kb was selected and purified using pulse field gel electrophoresis and electroelution techniques. The extracted DNA was separated by electrophoresis in a CHEF DRIII system (Bio-Rad, Hercules, CA, USA) in 0.5X Tris/borate/EDTA (TBE) at 14°C, using a 0.1 to 14 sec switch time at 6 V/cm for 12 h. The high molecular weight DNA (30 to 50 kb) was excised from the gel and recovered by electroelution techniques. The gel slice was electroeluted in a dialysis bag (Spectra/Por 4, Spectrum Laboratories, Rancho Dominguez, CA, USA) using a field strength of 70 V at 4°C for 2 h. The purified DNA solution was collected and subsequently concentrated using an Amicon Ultra filter unit (Millipore, Billerica, MA, USA).

### Fosmid library construction

A metagenomic fosmid library from the bagasse sample was constructed using a CopyControl™ Fosmid Library Production Kit (Epicentre Biotechnologies, Madison, WI, USA) according to the manufacturer’s instructions, with slight modifications. The purified DNA was end-repaired to generate blunt 5’-phospholyrated ends and then ligated to the pCC1FOS vector at 25°C for 3 h. The ligated DNA was packaged using the lambda packaging extract supplied and subsequently transformed into *Escherichia coli* EPI300-T1R. The transformants were selected on LB agar plates supplemented with 12.5 μg/ml of chloramphenicol. The library was stored at -80°C in 15% glycerol in the form of individual clones as well as pool libraries.

### Shotgun pyrosequencing and data pre-processing

A total of 3,300 randomly selected fosmid clones were sequenced on one full lane of the 454 GS-FLX Genome Sequencer System using the Titanium platform (Roche, Brandford, CT, USA) following the manufacturer’s protocol. Repeats in raw sequenced reads obtained were removed using RepeatMasker (http://www.repeatmasker.org). The vector and host sequences were filtered by BLASTN, with an E-value cutoff of 1e-3. The filtered reads were assembled using the Newbler assembly software, developed by 454 Life Sciences (version 2.6, Roche). Non-overlapping fragment singletons were clustered using the CD-HIT software [58] to minimize redundant sequences. The overall process of metagenomic data preparation and analysis is summarized in Additional file 1: Figure S1. The entire sequences of the bagasse fosmid library have been deposited to the NCBI Sequence Read Archive (SRA), which can be accessed using the accession number: SRX493840.

### Functional gene annotation and metabolic pathway analysis

The taxonomic classifications were performed on assembled contigs and singletons using BLASTN against the NT database. The E-value cutoff was set to 1e-3, and the best BLAST hit was used to refer the taxonomic rank of each sequence. The non-redundant singletons and contigs were predicted for open reading frames (ORFs) by MetaGeneMark [59]. The Shannon diversity index was computed using mothur [60] on 16S rRNA sequences extracted by BLASTN against the NCBI 16S microbial database using E-value cutoff 1e-5 and a minimal alignment length of 50 bp. The functional annotation was initially performed by stand-alone BLAST on predicted ORFs against the Non-Redundant protein database (NR) [23] using an E-value cutoff of 1e-6. The BLAST results containing the best hits were subsequently processed using the Blast2GO program [61] to assign their functional gene contents and enzymes based on the Kyoto Encyclopedia of Genes and Genomes (KEGG) [48]. Orthologous genes were identified using Clusters of Orthologous Groups (COGs) [43]. The carbohydrate-active enzymes were predicted using BLAST against the CAZy database using an E-value cutoff of 1e-10.

### Comparative metagenomic analysis of lignocellulose- and non-lignocellulose-degrading sources

We compared our bagasse metagenome to publicly available metagenomic projects obtained from NCBI Whole Genome Shotgun (WGS) and Sequence Read Archive (SRA) projects [31]. All additional datasets obtained were reanalyzed using the same procedures as described previously (as seen in Additional file 1: Figure S1) for an unbiased comparison. The publicly available datasets for lignocellulose-degrading and non-lignocellulose-degrading environments used in this study include metagenomic profiles from carcass [14], compost [32], farm soil [14], fresh water [62], human distal gut [33], marine water, mouse gut [39], peat swamp forest [15], sludge [34], termite gut [19], and wallaby gut [18] (summarized in Additional file 3: Table S2). Taxonomic distributions and functional genomic profiles of each metagenomic dataset are presented as unique hits (that is species or functional categories) or relative abundances, which are read counts normalized by the total number of mapped reads in each particular metagenome. Enrichment of read abundances was assessed using Fisher’s exact test against the all-combined dataset of the 12 metagenomes, or lignocellulolytic and non-lignocellulolytic groups. The *P*-values from Fisher’s exact test and odds ratios were derived using the module SciPy in Python (http://www.scipy.org/).

## Additional files

Additional file 1: Figure S1. Summary of data analyses and comparisons. The bagasse metagenomic fosmid library was pyrosequenced, the vector and host sequences removed, and it was assembled and deposited to the NCBI Sequence Read Archive (SRA). Additional metagenomic libraries of both lignocellulosic and non-lignocellulosic sources and their SRAs were obtained, and the subsequent functional analyses were performed using the same procedures. Additional file 2: Table S1. Summary of the numbers of hits that reads from the bagasse metagenomes were matched to for different taxonomic classifications. Additional file 3: Table S2. Numbers of total and mapped reads in lignocellulolytic and non-lignocellulolytic metagenomes retrieved and analyzed in this study. Additional file 4: Table S3. Numbers and percentages of reads, mapped to different NCBI taxonomy, COG, KEGG, and CAZy terms. Additional file 5: Table S4. Enrichment of taxa, COGs, and CAZy families in different metagenomes, as compared to reference set (lignocellulolytic and non-lignocellulolytic groups and all-combined group). *P*-values were computed using Fisher’s exact test, together with odds ratios, as described in the last tab in the table. Additional file 6: Figure S2. Percentage of metagenomic sequences mapped to the KEGG pathways, relative to all the reads in each metagenomic dataset. Additional file 7: Table S5. Correlations based on similarity of taxonomy, COGs, KEGG, and CAZy between the lignocellulose-degrading environment group (C), non-lignocellulose-degrading group (N), or libraries from the two different groups.

## Abbreviations

AA: auxiliary activity
bp: base pairs
CAZy: Carbohydrate-Active enZYmes
CBM: carbohydrate-binding module
COGs: Clusters of Orthologous Groups
GH: glycoside hydrolase
KEGG: Kyoto Encyclopedia of Genes and Genomes
ORF: open reading frame
PCA: principal component analysis
SD: standard deviation
SDS: sodium dodecyl sulfate
TCA cycle: tricarboxylic acid cycle
