# Supplementary figures and images for: Comparative analysis of sugarcane bagasse metagenome reveals unique and conserved biomass-degrading enzymes among lignocellulolytic microbial communities
Source: Biotechnol Biofuels. 2015 Feb 8;8:16. doi: 10.1186/s13068-015-0200-8 (PMC4337096; doi:10.1186/s13068-015-0200-8)

Figure S1

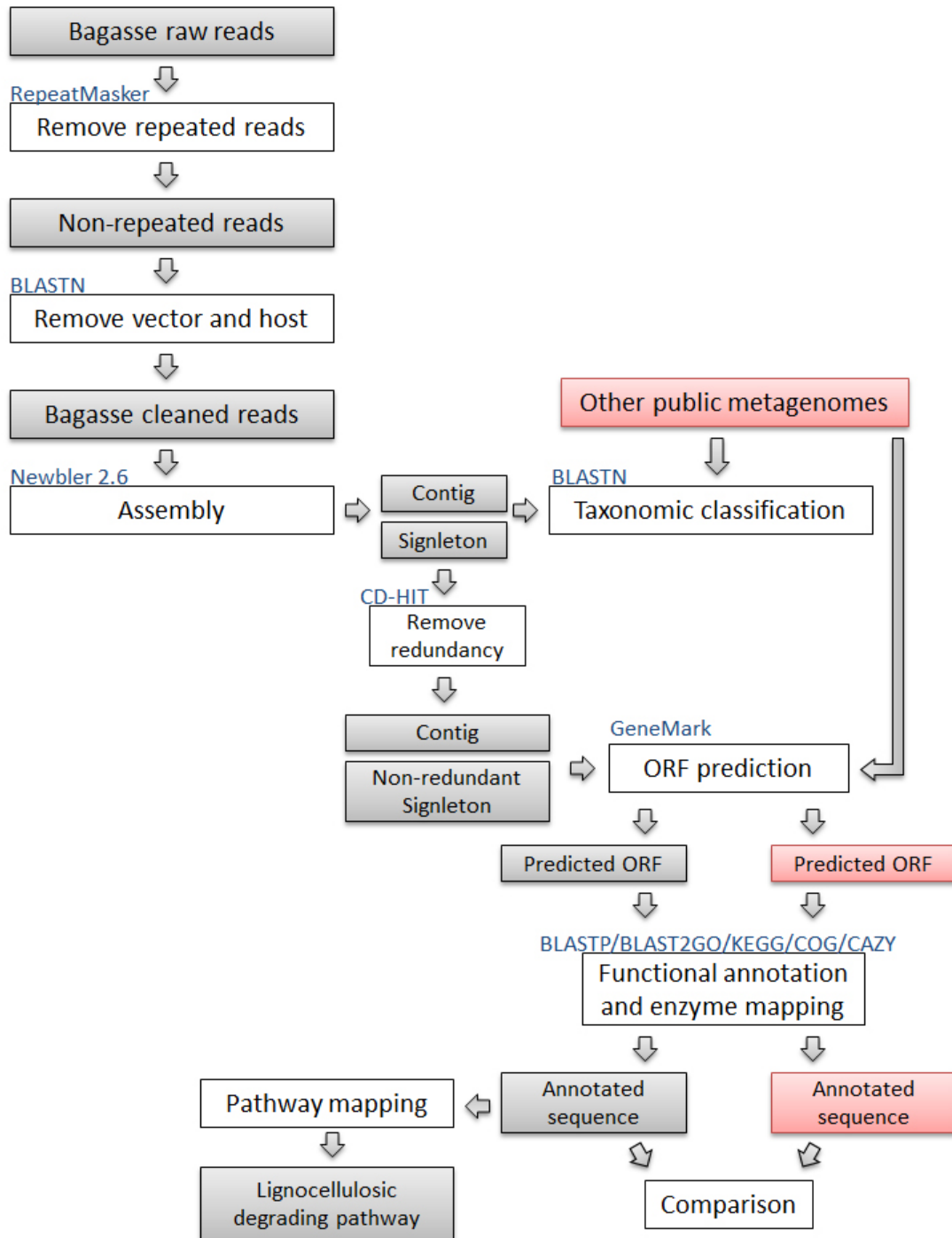

Supplement: Additional file 1: Figure S1. — Summary of data analyses and comparisons. The bagasse metagenomic fosmid library was pyrosequenced, the vector and host sequences removed, and it was assembled and deposited to the NCBI Sequence Read Archive (SRA). Additional metagenomic libraries of both lignocellulosic and non-lignocellulosic sources and their SRAs were obtained, and the subsequent functional analyses were performed using the same procedures. [file 13068_2015_200_MOESM1_ESM.pdf]

Figure S2

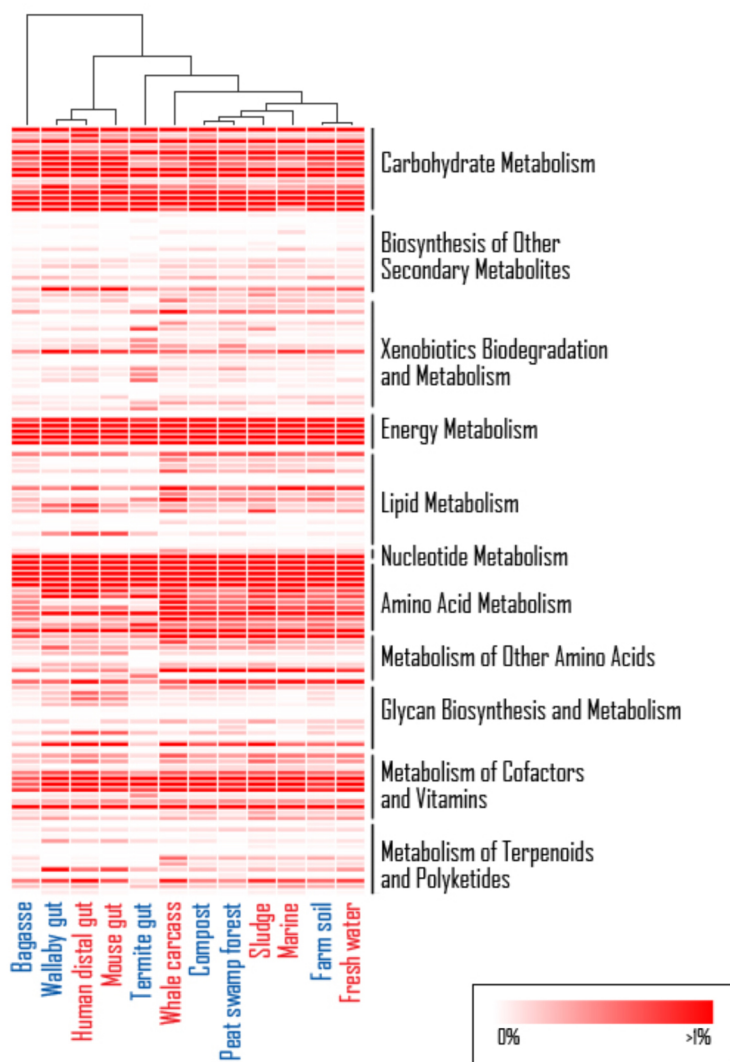

Supplement: Additional file 6: Figure S2. — Percentage of metagenomic sequences mapped to the KEGG pathways, relative to all the reads in each metagenomic dataset. [file 13068_2015_200_MOESM6_ESM.pdf]
