# Supplementary material for: Comparative analysis of sugarcane bagasse metagenome reveals unique and conserved biomass-degrading enzymes among lignocellulolytic microbial communities
Source: Biotechnol Biofuels. 2015 Feb 8;8:16. doi: 10.1186/s13068-015-0200-8 (PMC4337096; doi:10.1186/s13068-015-0200-8)
Supplement: Additional file 2: Table S1. — Summary of the numbers of hits that reads from the bagasse metagenomes were matched to for different taxonomic classifications. [file 13068_2015_200_MOESM2_ESM.docx]

**Supplementary Table 1: Taxonomic classification of organisms in bagasse fosmid library**

| **Taxonomic ranks** | | | | **NO. hit** | **Percentage** |
| --- | --- | --- | --- | --- | --- |
| Archaea | | | | 1,867 | 0.63 |
| Bacteria | Actinobacteria | Actinobacteridae | | 21,100 | 7.15 |
|  |  | Other | | 2,180 | 0.74 |
|  | Proteobacteria | Alphaproteobacteria | Rhizobiales | 34,740 | 11.78 |
|  |  |  | Rhodospirillales | 13,257 | 4.49 |
|  |  |  | Sphingomonadales | 7,737 | 2.62 |
|  |  |  | Caulobacterales | 6,403 | 2.17 |
|  |  |  | Other | 4,359 | 1.48 |
|  |  | Betaproteobacteria | Burkholderiales | 40,082 | 13.59 |
|  |  |  | Rhodocyclales | 8,960 | 3.04 |
|  |  |  | Other | 6329 | 2.15 |
|  |  | Deltaproteobacteria | Desulfovibrionales | 1,299 | 0.44 |
|  |  |  | Desulfuromonadales | 1,892 | 0.64 |
|  |  |  | Myxococcales | 12,307 | 4.17 |
|  |  |  | Other | 1,274 | 0.43 |
|  |  | Gammaproteobacteria | Enterobacteriales | 6,964 | 2.36 |
|  |  |  | Chromatiales | 14,408 | 4.88 |
|  |  |  | Xanthomonadales | 13,687 | 4.64 |
|  |  |  | Pseudomonadales | 11,478 | 3.89 |
|  |  |  | Methylococcales | 4,985 | 1.69 |
|  |  |  | Other | 4,709 | 1.60 |
|  |  | Other | | 4 | 0.00 |
|  | Bacteroidetes | Flavobacteriia | | 15,962 | 5.41 |
|  |  | Sphingobacteriia | | 6,288 | 2.13 |
|  |  | Other | | 6885 | 2.33 |
|  | Chlorobi | | | 943 | 0.32 |
|  | Acidobacteria | Acidobacteriales | | 2086 | 0.71 |
|  |  | Solibacterales | | 3457 | 1.17 |
|  |  | Other | | 3026 | 1.03 |
|  | Firmicutes | Bacilli | | 2,555 | 0.87 |
|  |  | Clostridia | | 3,064 | 1.04 |
|  |  | Other | | 428 | 0.15 |
|  | Chloroflexi | Chloroflexales | | 1,905 | 0.65 |
|  |  | Thermomicrobia | | 1,269 | 0.43 |
|  |  | Other | | 249 | 0.08 |
|  | Other bacteria | | | 12,189 | 4.13 |
| Eukaryota | Opisthokonta | | | 4,521 | 1.53 |
|  | Viridiplantae | | | 4,309 | 1.46 |
|  | Euglenozoa | | | 3,623 | 1.23 |
|  | Other Eukaryota | | | 312 | 0.11 |
| environmental samples | | | | 813 | 0.28 |
| ssRNA viruses | | | | 769 | 0.26 |
| dsDNA viruses, no RNA stage | | | | 305 | 0.10 |
| Unidentified and other sequences | | | | 44 | 0.01 |
| **Total** | | | | 295,023 | 100.00 |
